# Discovery of a new genus and species of dogielinotid amphipod (Crustacea: Amphipoda: Dogielinotidae) from the Nipa palm in Thailand, with an updated key to the genera

**DOI:** 10.1371/journal.pone.0204299

**Published:** 2018-10-15

**Authors:** Koraon Wongkamhaeng, Pongrat Dumrongrojwattana, Myung‐Hwa Shin

**Affiliations:** 1 Department of Zoology, Faculty of Science, Kasetsart University, Bangkok, Thailand; 2 Coastal Oceanography and Climate Change Research Center, Prince of Songkla University, Hatyai, Songkhla, Thailand; 3 Department of Biology, Faculty of Science, Burapha University, Bangsaen, Chonburi, Thailand; 4 National Marine Biodiversity Institute of Korea, Seocheon, South Korea; Nanjing Agricultural University, CHINA

## Abstract

During a scientific survey, a new genus of the dogielinotid amphipoda was found in the Nipa palm (*Nypa fruticans*) in Bang Krachao Urban Oasis, Samut Prakan Province, Thailand. We placed this new genus, *Allorchestoides* gen. nov., within the family Dogielinotidae. The new taxa can be easily distinguished from the remaining genera by differences in the incisor of the left and right mandibles, apical robust setae of the maxilla 1, and the large coxa and strong obtuse palm in the female gnathopod 1. The type species of *Allorchestoides* gen. nov., *Allorchestoides rosea* n. sp., is described here in, with an updated key to the genera of the family Dogielinotidae.

## Introduction

Talitroidea Rafinesque, 1815 is a member of the most well-known amphipod groups in terrestrial or semiterrestrial habitats. Taxonomic revisions on the status of the taxon have been carried out for a long time by many amphipodologists. The first revision of Bulycheva [1] elevated the status of the family Talitridae to the superfamily Talitroidea. As a result of the continuous revisions by Barnard and Karaman [2], Bousfield [3], Bousfield and Hendrycks [4], Serejo [5] and Lowry and Myers [6], the superfamily Talitroidea is currently composed of 11 families: Ceinidae J.L. Barnard, 1972; Chiltoniidae J.L. Barnard, 1972; Dogielinotidae Gurjanova, 1953; Eophliantidae Sheard, 1936; Hyalellidae Bulyčeva, 1957; Hyalidae Bulyčeva, 1957; Najnidae J.L. Barnard, 1972; Phliantidae Stebbing, 1899; Plioplateidae J.L. Barnard, 1978; Talitridae Rafinesque, 1815; and Temnophliantidae Griffiths, 1975.

The family Dogielinotidae is distributed in the temperate zones in the Pacific Ocean and tropical zones in the Indian Ocean in shallow water beaches (Derjavin [7]; Kamihira [8]; Barnard & Karaman [3]; Lazo-Wasem & Gable [9]; Odelvig [10]; Serejo [5]; and Bousfield [11]). The family was established by Gurjanova [12] in 1953, together with the monotypic genus *Dogielinotus*, based on the species *Dogielinotus moskvitini* (Derzhavin, 1930). In 2004, Serejo [5] divided the family Dogielinotidae into three subfamilies: Dogielinotinae, Hyalellinae, and Najaninae. Recently, a revision by Lowry and Myers in 2013 [6] raised these subfamilies to the family level.

Until now, 37 species belonging to 10 genera have been reported in the family Dogielinotidae [13]. During a scientific survey, however, a new member of the family Dogielinotidae was discovered in the Nipa palm (*Nypa fruticans*) in Bang Krachao Urban Oasis, Samut Prakan Province, Thailand, and it is not assignable to any previously known genera of Dogielinotidae. In the present study, we describe and illustrate a new genus and a new species.

## Material and methods

**Fig 1:**
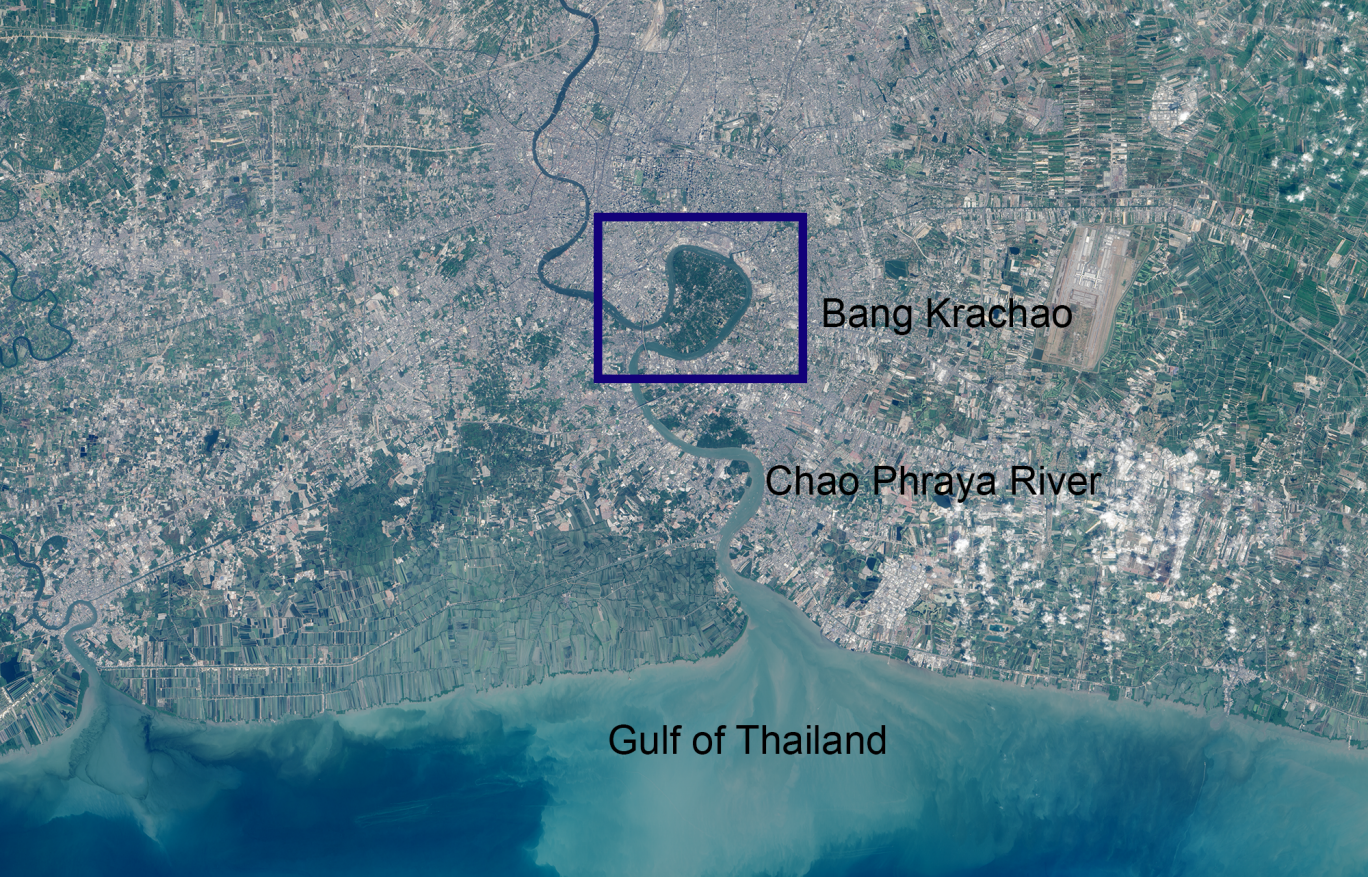
Map of sampling area.

Amphipods were collected from Nipa palm leaves in the Bang Krachao Estuary, near the mouth of the Chao Phraya River mouth (13°41'47.4"N 100°33'52.4"E) (Fig 1). The leaves were torn, and the amphipod specimens inside were sorted out and fixed in formalin for one week; then, they were stored in 70% ethanol. In the laboratory, the specimens were transferred from ethanol into glycerol for morphological study. Drawings were made using a drawing tube attached to an Olympus CH30 light microscope. The pencil drawings were scanned and digitally inked using a WACOM bamboo CTH-970 graphics board via Adobe Illustrator CC 2017, following the method described in Coleman [14]. The setae description was following the work of Garm [15]. The specimens were deposited in the Prince of Songkla University Zoological Collection (PSUZC).

## Ethics statement

This study was carried out in strict accordance with the recommendations in Animal Care & Use Guidelines of Institutional Animal Care and Use Committee of Burapha University (IACUC BUU). Amphipods were prior anesthetized with ice, then fixed in 10% formalin and finally preserved in 70% ethanol. The study area is a public area which no specific permissions were required for this location. All efforts were made to minimize suffering and habitat destruction. This work did not involve endangered or protected species.

## Nomenclatural acts

The electronic edition of this article conforms to the requirements of the amended International Code of Zoological Nomen clature, and hence the new names contained herein are available under that Code from the electronic edition of this article. This published work and the nomenclatural acts it contains have been registered in ZooBank, the online registration system for the ICZN. The ZooBank LSIDs (Life Science Identifiers) can be resolved and the associated information viewed through any standard web browser by appending the LSID to the prefix "http://zoobank.org/". The LSID for this publication is: urn:lsid:zoobank.org:pub:6313CE72-9F23-47C6-9724-879AF0E570B0. The electronic edition of this work was published in a journal with an ISSN, and has been archived and is available from the following digital repositories: PubMed Central, LOCKSS.

## Results

Order Amphipoda Latreille, 1816

Suborder Senticaudata Lowry & Myers, 2013

Family Dogielinotidae Gurjanova, 1953

***Allorchestoides* gen. n.** *urn*:*lsid*:*zoobank*.*org*:*pub*:*6313CE72-9F23-47C6-9724-879AF0E570B0*

**Diagnosis:** Male. Mouthparts, mandible, right incisor process four dentate; left incisor process six dentate; accessory setal row present; molar triturative. Maxilla 1 outer plate with six distal setal-teeth. Maxilla 2, inner plate with an enlarged proximal seta; outer plate subequal to inner plate in length. Maxilliped, outer plate shorter than article 2 of maxilliped palp; palp well-developed, dactyl unguiform. Coxal plates 1–4 deep, subrectangular; coxal plate 1–3 posterior marginal cusp absent. Gnathopods sexually dimorphic. Male gnathopod 1 weakly chelate; carpal lobe well-developed; palm slightly protruding at palmar corner, dactylus fitting palm. Gnathopod 2 propodal palm smoothly concave, interior margin lined with pappose setae. Epimeral side plates ordinary, plate 2 deepest. Pleopods peduncle with 2 small retinacula;

Female. Gnathopods 1 and 2 weakly chelate; carpal lobe well-developed, surpassing over propodus.

**Type species:** *Allorchestoides rosea*, new species, here designated.

**Etymology**: The specific name, *Allorchestoides*, alludes to fact that the new genus is allied to *Allorchestes* Dana, 1849. The gender is feminine as the gender adopted by its original authors.

**Remarks:** The new genus is similar to *Allorchestes* Dana, 1849, from the north and south Pacific [14], because it has a dactylus of maxilliped unguiform; carpus of male gnathopod 2 lobate, projecting between the merus and propodus; uropod 3 uniramus; and telson cleft that is half-length. However, the 1-articulate maxilla 1 palp in *Allorchestes* is reduced and tiny, not reaching the base of the setal-teeth of the outer lobe, while that of *Allorchestoides* gen. n. is absent (Table 1).

**Table 1:** Comparison of diagnostic character in dogielinotid genera.

**Genera**	**Antenna 2 sexual dimorphism**	**Left lacinia mobilis**	**Maxilla 1 palp**	**carpus of male** **gnathopod 2**	**Article 4 of pereopods 5–6**	**Pereopods 3–7**	**U3 rami**	**Telson type**
***Allorchestes* Dana, 1849**	Present	six teeth	reduced, not reaching the base of the setal-teeth of the outer lobe	reduced, posterior carpal lobe present	not expanded	with many stout and slender setae	uniramus	cleft until half of length or less
***Dogielinoides* Bousfield, 1982**	Absent	five teeth	reduced, not reaching the base of the setal-teeth of the outer lobe	reduced, posterior carpal lobe present	expanded	with many stout setae.	uniramus	cleft until half of length or less
***Dogielinotus* Gurjanova, 1953**	Present	?	reduced, not reaching the base of the setal-teeth of the outer lobe	well-developed, posterior carpal lobe present	expanded	with few stout setae	uniramus	cleft until half of length or less
***Eohaustorioides* Bousfield in Bousfield & Tzvetkova, 1982**	Absent	five teeth	reduced, not reaching the base of the setal-teeth of the outer lobe	well-developed, posterior carpal lobe present	expanded	with many stout setae.	uniramus	cleft to the base
***Exhyalella* Stebbing, 1917**	Present	five teeth	absent	reduced, posterior carpal lobe absent	not expanded	with few stout setae	uniramus	entire.
***Haustorioides* Oldevig, 1958**	Absent	five teeth	reduced, not reaching the base of the setal-teeth of the outer lobe	well-developed, posterior carpal lobe present	expanded	with many stout setae.	lacking rami	entire.
**Genera**	**Antenna 2 sexual dimorphism**	**Left lacinia mobilis**	**Maxilla 1 palp**	**carpus of male** **gnathopod 2**	**Article 4 of pereopods 5–6**	**Pereopods 3–7**	**U3 rami**	**Telson type**
***Insula* Kunkel, 1910**	Absent	?	reduced, not reaching the base of the setal-teeth of the outer lobe	well-developed, posterior carpal lobe absent	?	?	uniramus	entire.
***Marinohyalella*** **Lazo-Wasem & Gable, 2001**	Present	five teeth	reduced, not reaching the base of the setal-teeth of the outer lobe	reduced, posterior carpal lobe present	expanded	with few stout setae	uniramus	entire.
***Parhyalella* Kunkel, 1910**	Absent	five teeth	absent	reduced, posterior carpal lobe absent	not expanded	with few stout setae	outer ramus developed, inner ramus not reaching half of outer ramus	cleft to the base
***Proboscinotus* Bousfield, 1982**	Absent	five teeh	reduced, not reaching the base of the setal-teeth of the outer lobe	reduced, posterior carpal lobe present	expanded	with many stout and slender setae	uniramus	cleft until half of length or less
***Allorchestoides* nov.gen.**	Absent	four teeth	absent	well-developed, posterior carpal lobe present	not expanded	with few stout setae	uniramus	cleft until half of length or less

The *Allorchestoides* gen. n. shares some characteristics with *Exhyalella* Stebbing, 1917, from the Indian Ocean, because it has a vestigial maxilla 1 palp and uropod 3 uniramus. However, the current genus differs from *Exhyalella* because the carpus of the male gnathopod 2 is well-developed, the posterior carpal lobe is present (vs. carpus reduced, posterior carpal lobe absent), and the telson cleft is half-length (vs. entire) (Table 1).

The new genus can be easily distinguished from the remaining genera: 1) its right and left mandible has a 4-toothed and 6-toothed incisor, respectively; 2) the outer plate of the maxilla 1 contains 6 apical robust setal-teeth, whereas other talitroideans contain 9 apical robust setal-teeth; and 3) the peduncle and ramus of the uropod 3 are lined with robust setae and pappose setae. Moreover, the female of this new genus is characterized by having a large coxa 1 strongly produced posterodistally, and a gnathopod 1 propodus palm strongly obtuse with long oostegites and an apex sharply rounded with sparse setae.

The amphipod species is categorized into Dogielinotidae Gurjanova, 1953, as 1) antenna 1 is longer than the peduncle of antenna 2, 2) it has a mandibular molar palp vestigial and molar trituative, 3) article 4 of the maxilliped palp is well-developed, and 4) the male gnathopod 2 exhibits sexually dimorphic and a telson clef that is half of the length.

The new genus could be identified using the following updated key to include *Allorchestoides* gen. n.

Key to the genera of Dogielinotidae

1. Telson cleft to the base...................................................................................... 2
  Telson cleft until half length or less........................................................................... 3
  Telson entire........................................................................................................... 7
2(1). Palp of maxilla1 reduced, not reaching the base of the setal-teeth of the outer lobe; Coxae 2–3 1.5 times wider than long; Carpus of male gnathopod 2 triangular, well developed; Pereopod 3–7 with many stout setae.................................. ***Eohaustorioides***
  Palp of maxilla1 vestigial; Coxae 2–3 about as long as wide; Carpus of male gnathopod 2 triangular, reduced; Pereopod 3–7 with few stout setae ***Parhyalella***
3(1). Sexual dimorphism in peduncle of A2 absent................................................ 4
  Sexual dimorphism in peduncle of A2 present....................................................... 6
4(3). Left lacinia mobilis five teeth; Coxae 2–3 1.5 times longer than wide; Carpus of male gnathopod 2 triangular, reduced...................................................................... 5
  Left lacinia mobilis four teeth; Coxae 2–3 1.5 times wider than long; Carpus of male gnathopod 2 triangular, welldeveloped................................. ***Allorchestoides* nov.gen.**
5(4). Carpus of male gnathopod 2 posterior carpal lobe absent....... ***Dogielinoides***
  Carpus of male gnathopod 2 posterior carpal lobe present................ ***Proboscinotus***
6(3). Carpus of male gnathopod 2 triangular, well developed; Article 4 of pereopods 5–6 expanded................................................................................................ ***Dogielinotus***
  Carpus of male gnathopod 2 triangular, reduced; Article 4 of pereopods 5–6 not expanded.................................................................................................. ***Allorchestes***
7(1). Carpus of male gnathopod 2 triangular, well developed 8
  Carpus of male gnathopod 2 triangular, reduced.......................................... ***Exhyalella***
  Carpus of male gnathopod 2 rectangular, reduced................................... ***Marinohyalella***
8(7). Carpus of male gnathopod 2 posterior carpal lobe absent; Coxae 2–3 1.5 times longer than wide; Uropod 3 uniramus ..........................................................................................................***Insula***
  Carpus of male gnathopod 2 posterior carpal lobe present; Size/length ratio of coxae 2–3 1.5 times wider than long; Uropod 3 lacking rami ***Haustorioides***

***Allorchestoides rosea* n. sp.** *urn*:*lsid*:*zoobank*.*org*:*act*:*0EC10A22-C4A5-4997-8970-E81CE8261B1D*

**Fig 2:**
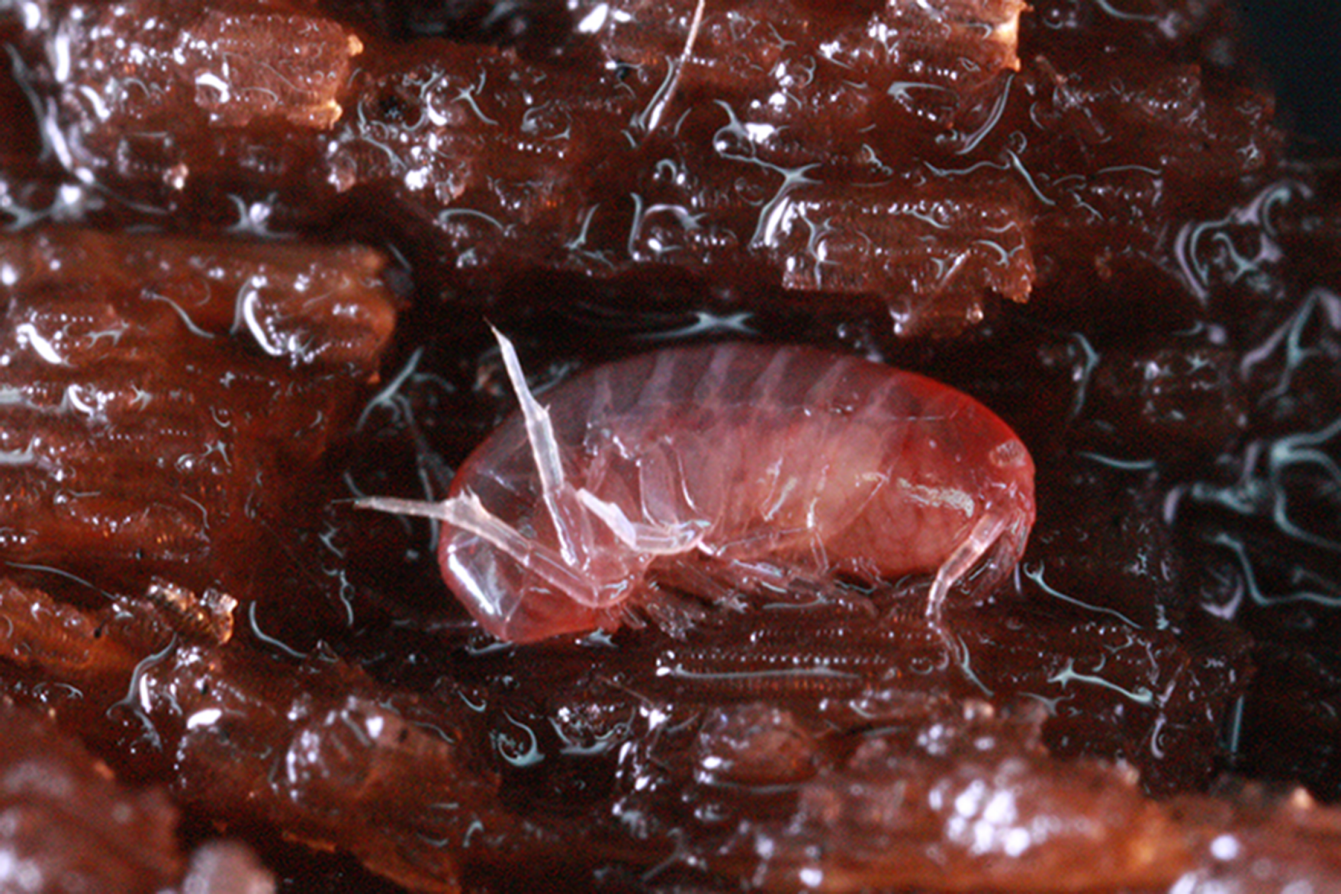
*Allorchestoides rosea* sp. n., holotype, male, (PSUZC-CR-00300).

**Etymology:** This species is named after the distinct reddish color while the amphipod alive (Fig 2).

**Type locality:** THAILAND, Bang Krachao Estuary near Chao Phraya River mouth (13°41'47.4"N 100°33'52.4"E), Nipa Palm leafs in mangrove forest, 2016, Dumrongrojwattana,P.

**Type material:** Holotype. ♂, PSUZC-CR-0300. Allotype, ♀ collected with holotype;

PSUZC-CR-0301; Paratype, collected with holotype (PSUZC-CR-302 (5♂; 5♀)).

**Diagnosis:** Same as the genus

**Fig 3:**
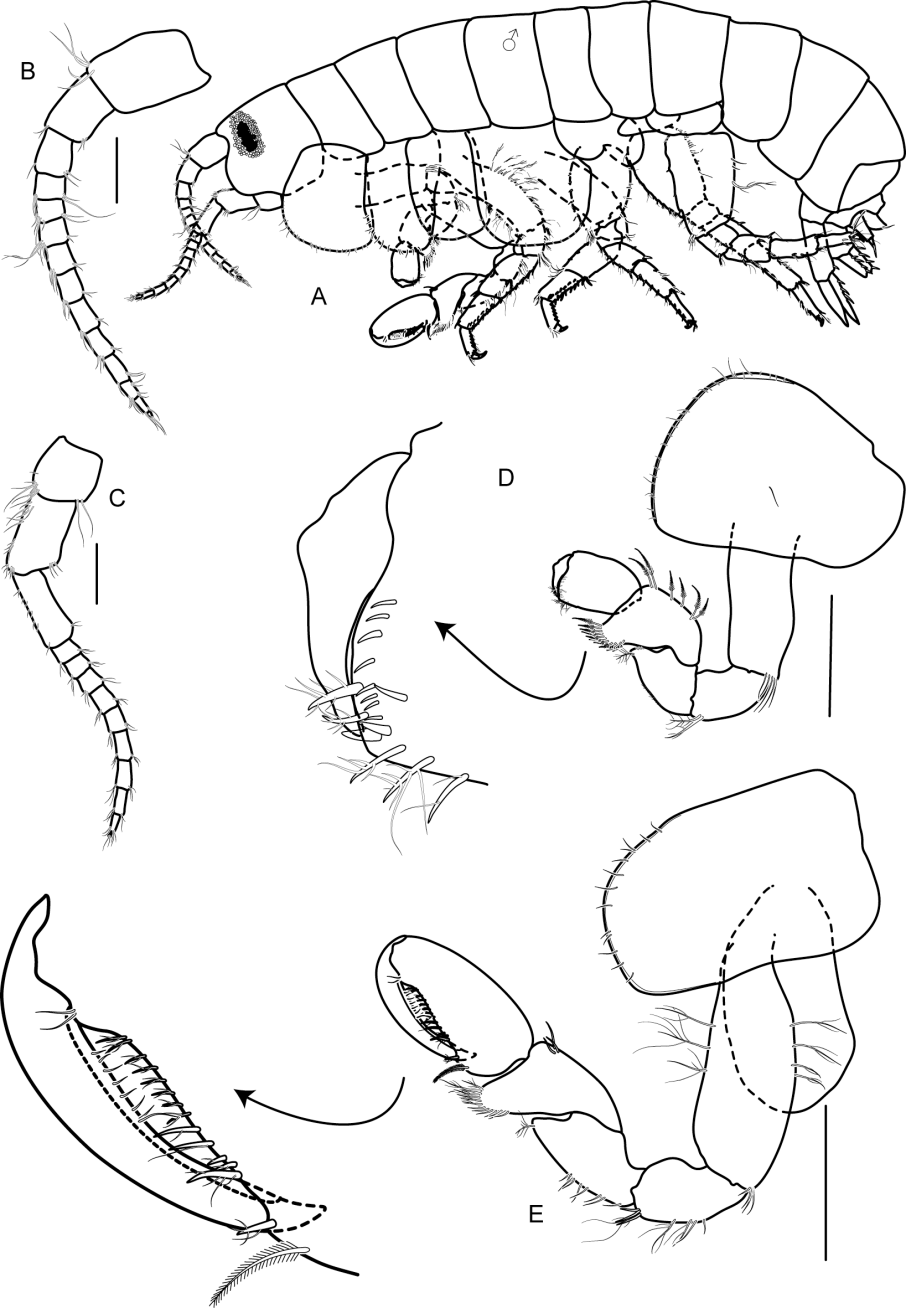
*Allorchestoides rosea* sp. n. holotype male (PSUZC-CR-00300). (A) male body, lateral (PSUZC-CR-00300), (B) antenna 1, (C) antenna 2, (D) gnathopod 1, (E) gnathopod 2. Scales bars: 0.5 mm.

**Description**: Based on holotype male 6.6 mm. Body (Fig 3A), medium, smooth.

**Fig 4:**
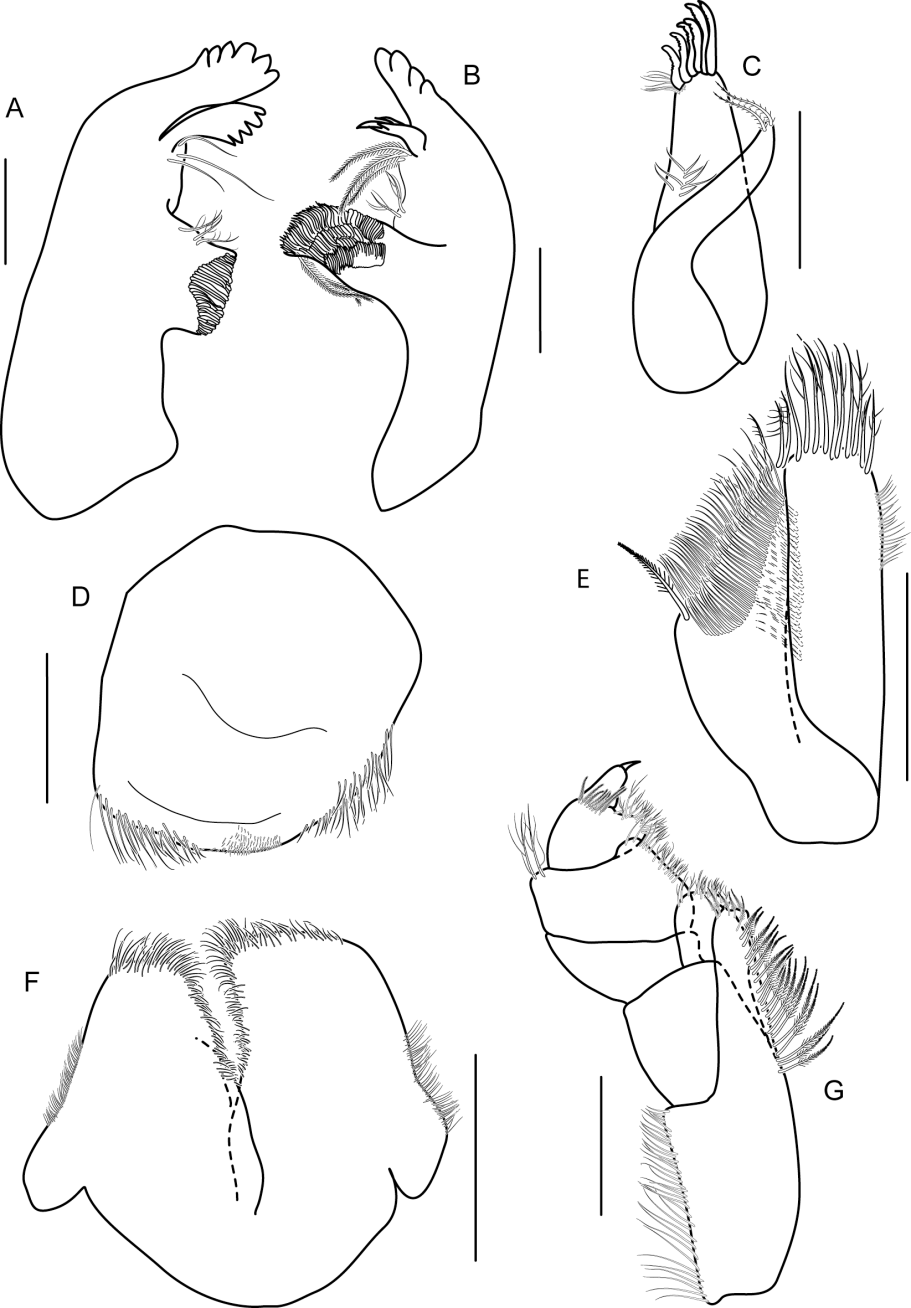
*Allorchestoides rosea* sp. n. holotype male (PSUZC-CR-00300). (A) left mandible, (B) right mandible, (C) maxilla 1, (D) upper lip, (E) maxilla 2, (F) lower lip, (G) maxilliped. Scales bars: 0.2 mm.

Head. Eye medium (1/3 head length), subreniform, black. Antenna 1 (Fig 3B) long, subequal to antenna 2, flagellum of 11 short articles of flagellum, lined with pappose setae. Antenna 2 (Fig 3C) peduncular articles slender, article 5 subeaqual to article 4; flagellum of 11 articles, longer than peduncles, final article minute. Upper lip (Fig 4D) broad, deep, apex rounded. Lower lip (Fig 4F) without inner plates; outer plate with rounded lateroapical margin. Right mandible (Fig 4B) incisor 4-dentate, lacinia mobilis 5-dentate. Left mandible (Fig 4A) incisor 6-dentate, lacinia mobilis 4-dentate, molar triturative, strong. Maxilla 1 (Fig 4C) inner plate rod-shaped with two terminal setae; outer plate with six terminal serrate setal teeth, palp absent. Maxilla 2 (Fig 4E) inner plate triangular-shape lined with pappose setae, subequal to outer plate in length. Maxilliped (Fig 4G) inner plate slender, with apical and subapical plumose setae and two large conical robust setae; outer plate with apical pappose setae; palp article 2 distomedial lobe well-developed; article 4 long, rod-shaped, unguiform.

Pereon. Gnathopod 1 (Fig 3D); weakly chelate, sexually dimorphic; coxa subequal to coxa 2 in length, ventral margin lined with short setae; carpus longer than propodus, triangular, well-developed with posterior carpal lobe posterior margin with pappose setae; propodus oval, palm transverse, fringed with eight short robust setae, four robust setae and 1 anterodistal seta; dactylus fitting with palm, anteroproximal margin protruded.

Gnathopod 2 (Fig 3E); larger than gnathopod 1, subchelate, sexually dimorphic; coxa subrectangular, longer than broad; basis lined with marginal pappose setae; ischium and merus with posterior marginal pappose setae; carpus triangular, well-developed, posterodistal lobe produced, rounded, beset with pappose setae; propodus ovate, two times as long as wide; palm acute, excavated, longer than posterior margin, lined with pappose setae; dactylus strongly curved, extending 33% along posterior margin, lined with setae, fit with palm.

**Fig 5:**
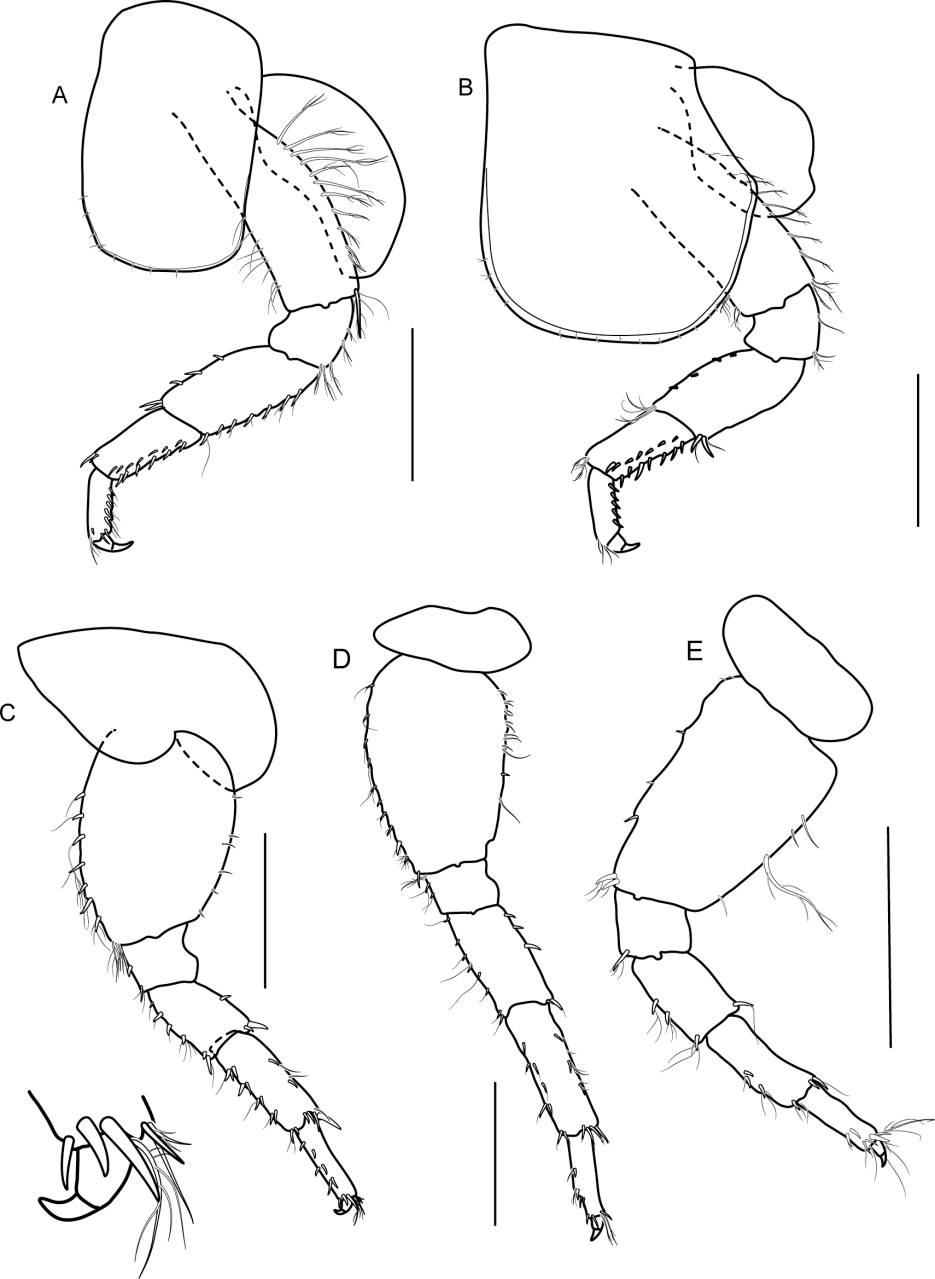
*Allorchestoides rosea* sp. n. holotype male (PSUZC-CR-00300). (A) pereopod 3, (B) pereopod 4, (C) pereopod 5, (D) pereopod 6, (E) pereopod 7. Scales bars: 0.5 mm.

Pereopods 3–4 (Fig 5A and 5B) alike, subequal, coxae subquadrate, coxa 4 with posterior lobe, merus longer than carpus, not expanded, fringed with robust setae; carpus fringed with two rows of posterior marginal robust setae; propodus slender, prehensile shorter than carpus, with posterior marginal robust setae; dactylus short, curved. Pereopod 5 (Fig 5C) coxa bilobed; anterior of basis to propodus fringed with robust setae; merus not expanded, propodus prehensile, slightly shorter than carpus, with three terminal clasping robust setae; dactylus without inner marginal seta. Pereopods 6 and 7 (Fig 5D and 5E) alike, subequal, coxae ovate; anterior of basis lined with robust setae; merus not expanded; carpus slightly longer than propodus; propodus weakly prehensile with three terminal clasping robust setae; dactylus short, curved (Fig 5C).

**Fig 6:**
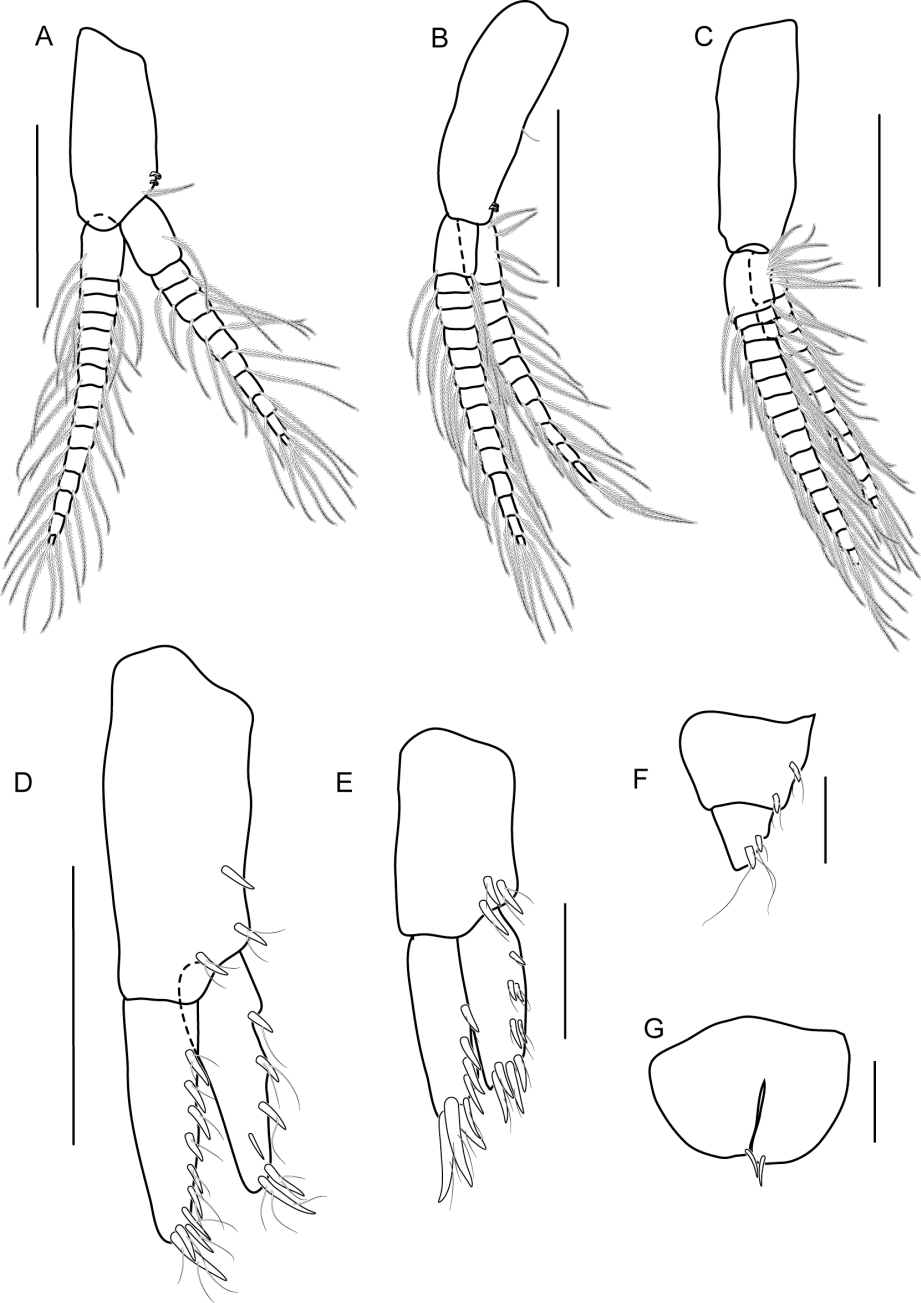
*Allorchestoides rosea* sp. n. holotype male (PSUZC-CR-00300). (A) pleopod 1, (B) pleopod 2, (C) pleopod 3, (D) uropod 1, (E) uropod 2, (F) uropod 3, (G) telson. Scale bars: A-D: 0.5 mm, E-G: 0.2 mm.

Pleopods 1–3 well-developed, peduncle longer than broad, without marginal setae; biramous, inner ramus slightly shorter than outer ramus. Epimera 1–3 round, subequal. Uropod 1 peduncle, two times as long as broad, with two robust setae; distolateral robust seta present. Uropod 2 peduncle, with three distal robust setae; inner ramus slightly shorter than outer ramus, with marginal robust setae and apical robust setae. Uropod 3 uniramus, not fused to peduncle; peduncle with two robust setae dorsally; subequal to peduncle in length, Telson broader than long, cleft until half of length, each lobe with an apical seta (Fig 6).

**Fig 7:**
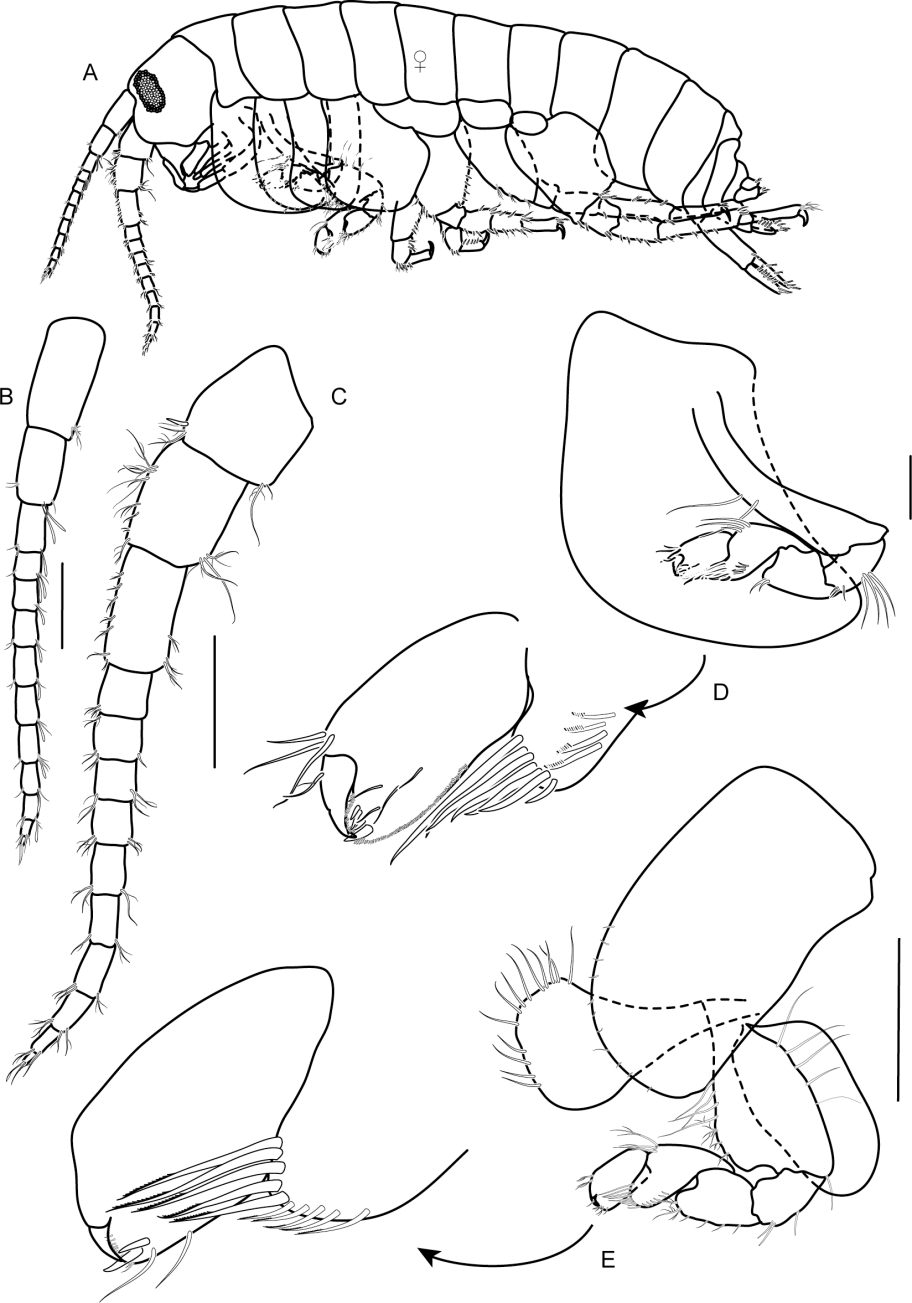
*Allorchestoides rosea* sp. n. Allotypes, female, (PSUZC-CR-00301). (A) Female body, lateral, (B) antenna 1, (C) antenna 2, (D) gnathopod 1, (E) gnathopod 2. Scales bars: 0.5 mm.

**Female**: Based on paratype female 4.7 mm (Fig 7A).

Antenna 1 and 2 similar to those of male (Fig 7C and 7D). Gnathopod 1 (Fig 7D) smaller than gnathopod 2, chelate; coxa large, trapezoid, posteroventral margin expanded, round, and covered by coxa 2; posterior margin excavated; basis long and slender; merus trapezoid; carpus triangular with well-developed, anterodistal corner and posterodistal corner beset with robust setae, posterodistal lobe produced, rounded, over-reaching propodus; propodus ovate, palm to half of hind margin covered with short setae; palm strongly obtuse, palmar corner with two defining robust seta; dactylus fitting palm.

Gnathopod 2 (Fig 7E) wealky chelate; coxa subrectangular; basis expanded anterodistally, two times longer than wide; merus trapezoid, posteriordistal corner produced; carpus triangular, with well-developed posterodistal lobe, anterodistal corner and posterodistal corner beset with robust setae; propodus subovate, posterior margin shoter than anterior one; palm obtuse, lined with short setae and two defining robust setae on palmar corner; dactylus fitting palm.

**Fig 8:**
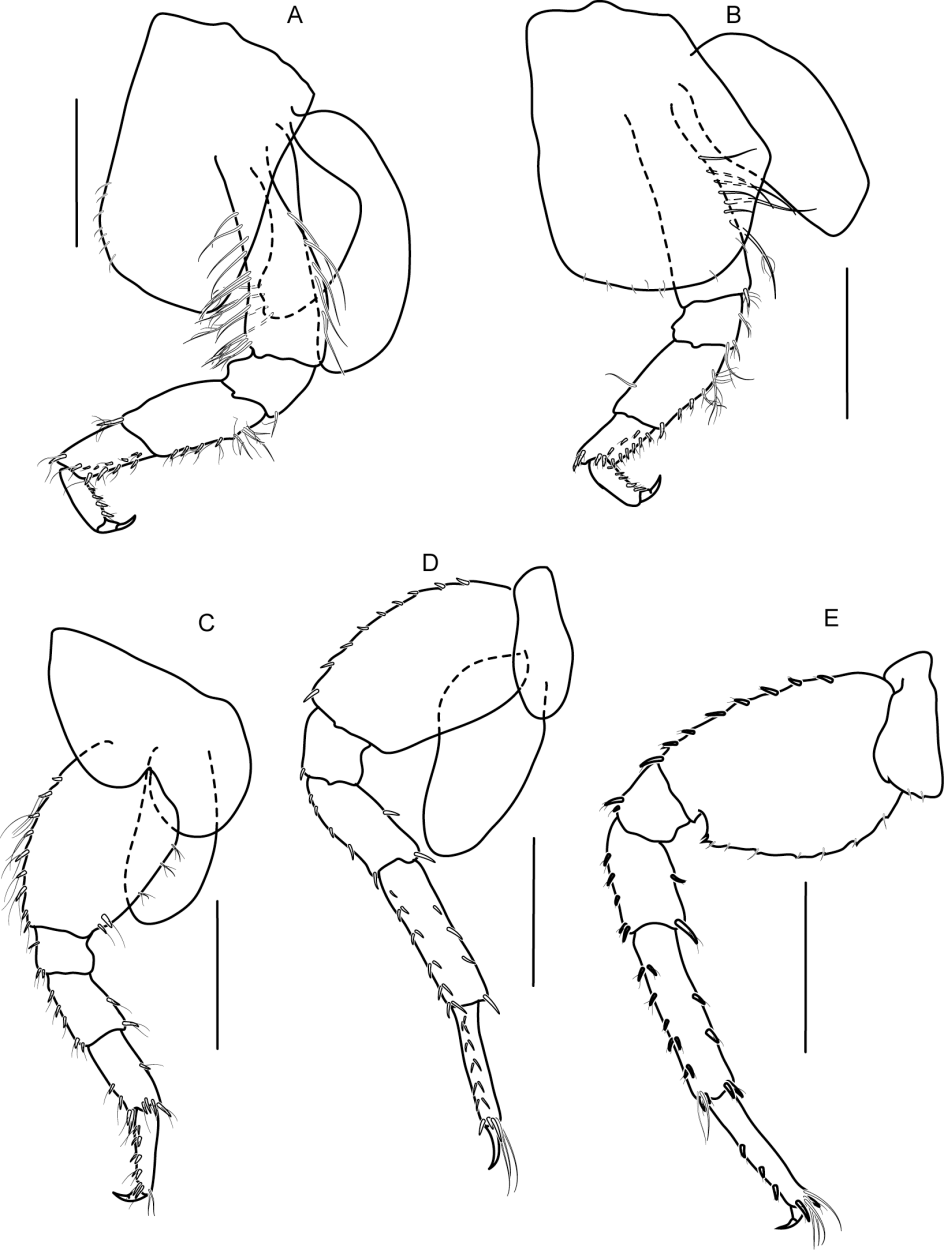
*Allorchestoides rosea* sp. n. Allotypes, female (PSUZC-CR-00301). (A) pereopod 3, (B) pereopod 4, (C) pereopod 5, (D) pereopod 6, (E) pereopod 7. Scales bars: 0.5 mm.

Oostegites on gnathopod 2 (Fig 7E) and pereopod 3 (Fig 8A) subequal, fringed with simple setae, large, broad, apex round.

## Discussion

All of the species of the family Dogielinotidae have been known to be marine members, and they are free-living in shallow water and shallow water and sand bottom [16]. The *Allorchestoides* gen. n. is the only genus in this group that lives in brackish water and is associated inside a submerged Nipa palm trunk.

Some modifications of their morphological characters would have them fit with the habitat of *Pseudamphithoides incurvaria* (Just, 1977), a species of house-building ampithoids amphipods that freely moves among hydroids and algae with their house by using prehensile pereopods 5–7 [17]. The prehensile dactyl of *Allorchestoides rosea* n. sp. might be used for grasping and moving among the fiber of the Nipa palm (S1 Video). Moreover, the pappose setae existing on the antennae, gnathopods, mandible, maxillae, maxilliped, and pereopods seem to be used for grooming food particles. Garm [15] described the pappose setae as the setae that contain long, well-developed setules scattered randomly along the total length of the shaft of the setae. The setae are always found laterally on the mouthparts, exopods, and endopods of the maxillae and maxilliped. That work focused on the setae that existed on the mouthparts and concluded that the pappose setae on the mouthparts of seven species of decapod function by acting as setal barriers, detecting current direction, and filtering. The setae were also present on the mandibular palp of the shrimp, *Penaeus monodon* Fabricius, 1798 where the roof of the feeding area was formed. In talitroidean amphipods, there is a variety of accessory grooming structures, i.e., setae, scale, and spine found on both gnathopods 1 and 2. In the aquatic group, the accessory grooming structure exist only on the gnathopod 1, and the gnathopod 2 is used for carrying [15, 18].

According to observations conducted in the laboratory, the amphipod feeds mainly on the palm tissue, which the food item present in stomach. The feeding procedure starts from the creation of the feeding current along the ventral part by pleopods, followed by grasping of the food items by the gnathopod 2 and grooming by the gnathopod 1 and antenna 2. The pappose setae occurring on antennae 1 and 2 and gnathopods 1 and 2 may be necessary for grooming to live among fiber of Nipa palm. Further investigations about the feeding behavior and feeding ecology of this group are needed to obtain a deeper understanding.

## Supporting information

S1 Video Amphipod feeding current. (MOV)
